# Geographic Variation in Resistance of the Invasive *Drosophila suzukii* to Parasitism by the Biological Control Agent, *Ganaspis brasiliensis*

**DOI:** 10.1111/eva.70043

**Published:** 2025-03-04

**Authors:** Oscar Istas, Marianna Szűcs

**Affiliations:** ^1^ Department of Entomology Michigan State University East Lansing Michigan USA

**Keywords:** biological control, geographic mosaic of coevolution, host–parasitoid coevolution, spotted wing drosophila

## Abstract

Host–parasitoid interactions are tied in coevolutionary arms races where parasitoids continuously have to evolve increased virulence as hosts evolve increased resistance. Over time, geographic structure in virulence and resistance can arise because of spatial and temporal differences in parasitoid communities, in the strength of reciprocal selection pressures, in genetic variation in local populations, and as trade‐offs are balanced between defense and fitness traits. It is crucial to understand the resistance structure of pest populations to successfully implement biological control programs against invasive insect hosts. We investigated spatial and temporal variations in the resistance of the invasive *Drosophila suzukii* in seven geographically distinct populations in Michigan and of one population from Oregon against a newly approved biocontrol agent, the larval parasitoid *Ganaspis brasiliensis.* We found regional and temporal variations in the resistance (encapsulation rates of parasitoid eggs) of *D. suzukii* populations that ranged from 11% to 48%. The northernmost, and thus the coldest site, had the highest rate of parasitism and the lowest encapsulation rate. Large regional differences in the resistance of *D. suzukii* populations can render the ensuing biocontrol program more variable and less predictable, and release strategies may need to be altered at sites where flies have high resistance.

## 1 Introduction

A multitude of interconnected invasion theories aim to explain the success and post‐introduction trait changes in invasive species (Catford, Jansson, and Nilsson 2009; Jeschke and Heger 2018). Most commonly, it is assumed that invasive species will experience some form of release from their enemies (Enemy Release Hypotheses—ERH) that will allow for their proliferation in the introduced range (Keane and Crawley 2002). As an extension of ERH, the Evolution of Increased Competitive Ability (EICA) hypothesis predicts that due to the release from specialist enemies, invasive species will need to allocate fewer resources toward often costly defenses, and in turn, those resources can be used to increase their growth and reproduction making them better competitors (Blossey and Notzold 1995; Honor and Colautti 2020). Instead of a reduction of defenses in invasive species, the Shifting Defense Hypothesis (SDH) predicts that the types of defenses deployed by invasive species may change as attack by coevolved specialist enemies decreases while attack by generalist enemies is sustained in the introduced range (Müller‐Schärer, Schaffner, and Steinger 2004; Joshi and Vrieling 2005). Host defenses can also be mediated by the residence time of invasive species as both generalist and specialist enemies will continue to accumulate on them (Cornell and Hawkins 1993; Strauss, Lau, and Carroll 2006; Stricker et al. 2016; Crous et al. 2017; Rodríguez et al. 2019) and as accidental introduction of specialist enemies restores some of the long‐term coevolutionary interactions (Rayamajhi et al. 2008; Wheeler et al. 2017; Miksanek and Heimpel 2019; Abram et al. 2022). Thus, invasive species may show decreased defenses as predicted by EICA, a shift in the type of defense used as predicted by SDH, or no change in defense compared to their native ranges depending on their residence time and the makeup of the natural enemy community in the invaded range. The above theories were developed for plant invasions, but their basic predictions should hold for invasive insects as well, that are commonly attacked by a suite of generalist and specialist natural enemies.

Herbivorous insects are tied in coevolutionary arms races with insect parasitoids that lay their eggs inside or on the host, and feeding by the developing larvae eventually kills the host (Godfray 1994; Kraaijeveld, van Alphen, and Godfray 1998; Thompson 2005a, 2005b). Since only one party survives this interaction, there is strong selection pressure for the prey to escape parasitism by mounting physiological and behavioral defenses (resistance) and for parasitoids to overcome host defenses and increase developmental success (virulence) (Godfray 1994). Both host resistance and parasitoid virulence can evolve rapidly (Jalvingh et al. 2014; Cavigliasso et al. 2019; Moiroux et al. 2018; Linder et al. 2022), and the maintenance of both can be costly (Kraaijeveld and Godfray 1997; Fellowes, Kraaijeveld, and Godfray 1998; Kraaijeveld, Limentani, and Godfray 2001; Kraaijeveld and Godfray 1999). In addition, there can be regional differences in virulence and resistance, as shown in European *Drosophila* species and their native parasitoids (Kraaijeveld, Nowee, and Najem 1995; Kraaijeveld and Godfray 1999). Geographic structure in resistance can arise because of spatial and temporal differences in the strength of reciprocal selection between hosts and parasitoids, differences in the genetic variation available in local populations to respond to selection, differences in the wider host–parasitoid community, in abiotic conditions, and how the cost and benefits of resistance are balanced locally in terms of fitness (Kraaijeveld and Van Alphen 1995; Kraaijeveld and Godfray 1999; Dubuffet et al. 2007). It is largely unknown how the coevolutionary dynamics of host–parasitoid interactions may change following the invasion of a new environment where initially mostly generalist parasitoids will attack the introduced insect hosts. It is also unclear whether invasive insect hosts would develop similar geographic variation in their defenses as native species despite their relatively short residence time and given that selection pressures by specialist parasitoids may be relaxed across the invaded range.

Invasive insect pests are often the target of classical biological control programs that focus on the introduction of specialized parasitoids from an invasive species' native range. As invasive hosts are reunited with their coevolved natural enemies, reciprocal selection pressures are restored, and a new coevolutionary arms race can ensue in the introduced range. The outcome of this new arms race, and thus the success or failure of the biological control program, can depend on the levels of defenses maintained by the introduced insect pests and encountered by the newly released parasitoids. Additionally, regional differences in host defenses can result in geographic variation in the impact of biological control agents. To better predict biocontrol efficacy, it is important to understand the resistance structure of pests in the introduced ranges.

The focus of this study is *Drosophila suzukii* (Matsumura) (Diptera: Drosophilidae) that invaded the Americas and Europe in 2008 (Asplen et al. 2015). *Drosophila suzukii* has a wide host range, attacking economically important crops such as raspberries, blueberries, strawberries, and cherries, as well as numerous wild hosts, such as dogwood, pokeweed, choke cherry, and elderberry (Asplen et al. 2015; Poyet et al. 2015; Lee et al. 2019). The extreme polyphagy coupled with high fecundity (> 600 eggs per female) and short generation times (~14 days at 22°C) make *D. suzukii* difficult to control (Wang et al. 2018). Furthermore, the large population sizes and multiple generations per season also provide ideal conditions for rapid evolution.

Since its invasion, *D. suzukii* has accumulated a diversity of generalist pupal parasitoids that are native in the introduced ranges. These parasitoids have had limited developmental success on *D. suzukii* (Chabert et al. 2012; Rossi‐Stacconi et al. 2015; Mazzetto et al. 2016; Knoll et al. 2017; Lee et al. 2019; Wolf, Baur, and Collatz 2019; Shaw et al. 2023). In North America, where this study took place, parasitism rates of *D. suzukii* remained under 10% for about a decade post‐introduction and were mostly limited to two native generalist pupal parasitoids (Lee et al. 2019). *Drosophila suzukii* was largely able to escape parasitism by native species in the introduced range because it has a stronger immune response than the local drosophilids (Kacsoh and Schlenke 2012; Poyet et al. 2013). It frequently encapsulates and kills the eggs and/or larvae of native larval parasitoids as it mounts a cellular and humoral immune response (Kacsoh and Schlenke 2012; Poyet et al. 2013). *Drosophila suzukii* has a high hemocyte load that is shown to correlate with increased levels of resistance to parasitism (McGonigle et al. 2017; Poyet et al. 2013). However, maintaining high levels of such constitutive resistance was shown to be costly in other *Drosophila* species and led to trade‐offs in the form of increased larval mortality and slower development in competitive environments and when food was limited (Kraaijeveld and Godfray 1997; Fellowes, Kraaijeveld, and Godfray 1998; Kraaijeveld, Limentani, and Godfray 2001; McGonigle et al. 2017). Parasitoids that could successfully and consistently develop in *D. suzukii* were first found in 2016 and in 2019 in British Columbia, Canada, in 2020 in the state of Washington, USA, and in 2019 in Europe with the unintentional introduction of two larval parasitoids, *Leptopilina japonica* (Novković & Kimura) (Hymenoptera: Figitidae) and *Ganaspis brasiliensis* (Ihering) (Hymenoptera: Figitidae), from the native ranges of *D. suzukii* in Asia (Abram et al. 2020; Puppato et al. 2020; Beers et al. 2022; Martin et al. 2023). By 2022, *L. japonica* has spread to 10 western, mid‐Atlantic, and Midwestern states in the USA, including Michigan. In addition, in 2022, field releases of the G1 strain of *G. brasiliensis* have begun across the USA as part of a new biological control program (Gariepy et al. 2024). Thus, *D. suzukii* existed without specialist natural enemies in the USA for about the first decade following its introduction with exposure to specialist parasitoids only in the last few years at the time of this study.

We investigated the spatial and temporal patterns of resistance of invasive *D. suzukii* populations by sampling seven geographically distinct populations in Michigan and one in Oregon over a 2‐month period in 2022. The aim was to assess the levels of resistance the specialist larval parasitoid *G. brasiliensis* may encounter following its widespread introduction as a biological control agent against *D. suzukii* and to determine if geographic variation exists in resistance that may mediate biocontrol success.

## 2 Materials and Methods

### 2.1 Parasitoid Rearing

We used the so‐called G1 strain of *G. brasiliensis* in our experiments that was originally collected from *D. suzukii* in Tokyo, Japan, in 2016 and transported to CABI‐Switzerland (Hopper et al. 2024). From this Swiss colony, 150 wasps were shipped to an Italian laboratory in 2020 (Girod et al. 2018; Hopper et al. 2024). The USDA‐ARS Beneficial Insects Introduction Research Unit in Newark, Delaware, received about 500 individuals of this Japanese colony via Italy in November 2021 (Hopper et al. 2024). All *G. brasiliensis* intentionally released in the USA originate from this laboratory colony that was reared for about three generations in Newark before redistribution across the states (W. Xingeng pers. comm.). Michigan State University (MSU) received a shipment of 50 adult males and 50 adult females in March 2022. The *G. brasiliensis* population used in our experiments was established using 15 adult males and 15 adult females from this MSU‐based colony in May 2022. Thus, this colony of *G. brasiliensis* spent about 6 years in culture prior to our experiments. Assuming one generation per month of *G. brasiliensis* in laboratory rearing, this means about 72 generations.

To rear *G. brasiliensis* , 150 g of store‐bought conventionally grown blueberries were placed into 25 × 19 × 25 cm plastic containers (PrepNaturals) and sprinkled with one teaspoon of instant dry yeast (Fleischmann's Active Dry Yeast) each to reduce mold buildup and ensure infestation from flies (Rossi‐Stacconi et al. 2022). Two containers were placed without lids in a 30 × 30 × 30 cm mesh cage (RestCloud), and 100 *D. suzukii* flies (50 males and 50 females) were released to infest the blueberries for 48 h. The *D. suzukii* flies used to infest the fruit originated from our laboratory colony that was founded in 2018 and augmented with wild‐caught individuals each year. The fly rearing proceeded as described by Jarrett et al. (2022) using the standard DSSC cornmeal diet in incubators set at 25°C ± 2°C, 70% ± 5% relative humidity, and a 16L:8D photoperiod.

The containers with the fly‐infested blueberries were then removed from the cages, and 30 *G. brasiliensis* individuals (15 male and 15 female) were added to each to parasitize *D. suzukii* larvae for 5 days. During parasitism, the containers were covered with lids that had fine mesh windows for ventilation and a strip of honey underneath for the adult parasitoids to feed on. The parasitoids were removed after 5 days and reused for a next round of parasitism of a new batch of *D. suzukii‐*infested blueberries. This process was repeated three to four times until most adult parasitoids died to maximize population growth. Following parasitism, the plastic containers were held in the same incubators used for fly rearing and were checked twice a week to remove any emerging *D. suzukii*. After approximately 28–35 days, *G. brasiliensis* adults emerged, which were then used to start the next parasitoid generation.

### 2.2 Field Sampling of *Drosophila suzukii* Populations

**FIGURE 1:**
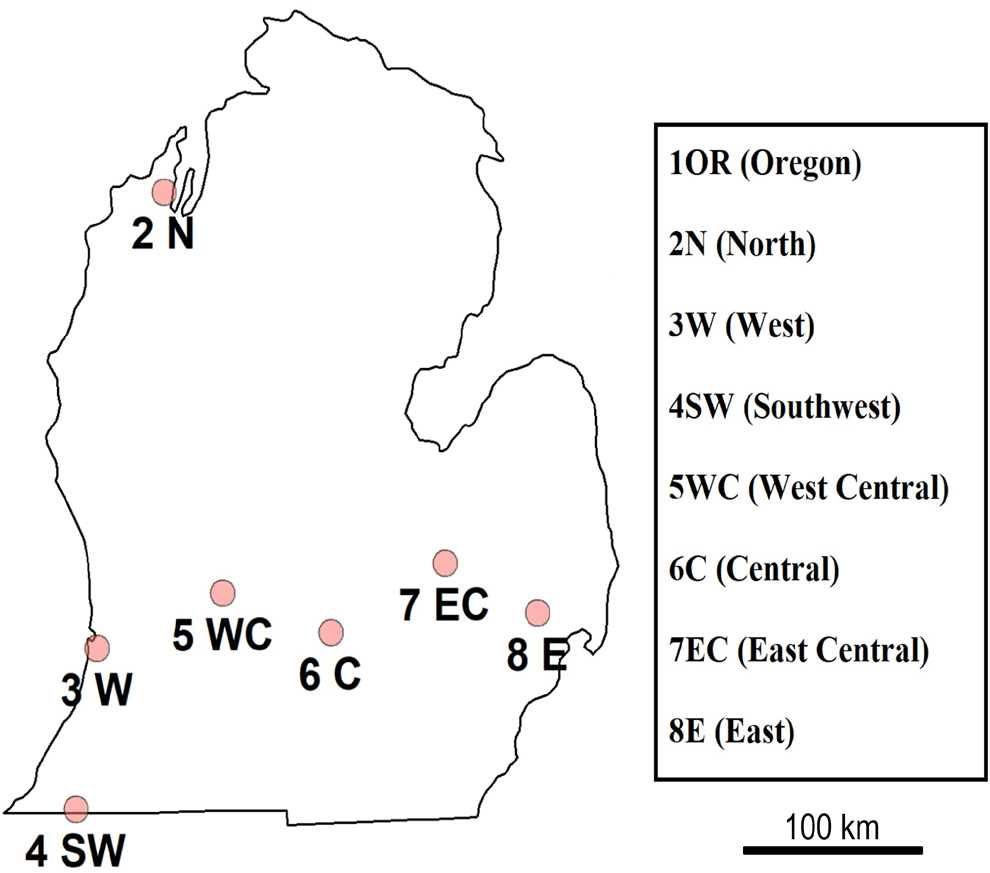
Sites sampled and assayed for *Drosophila suzukii* resistance in 2022. An additional location was sampled in Corvallis, OR. For coordinates, site and sampling information, see Table 1.

We sampled seven *D. suzukii* populations in Michigan in 2022 following a north‐to‐south and east‐to‐west grid (Figure 1) and received samples from one location in Corvallis, Oregon. The Oregon site was included in the sampling to represent a location from the western USA. It is also a neighboring state to California where *D. suzukii* was first detected (Asplen et al. 2015) and thus may represent a more direct dispersal route for the originally introduced *D. suzukii* genotypes than the populations from Michigan. The distance between the northernmost and southernmost field sites in Michigan was 400 km, and the westernmost and easternmost sites were about 320 km apart. Most sites were conventionally managed mixed orchards, while the site in Oregon and one site in Michigan were organically managed mixed fruit and vegetable farms (Table 1). *Drosophila suzukii* was collected from each location once in August and in September. At each date, three types of fruit samples were collected: (1) managed fruit (cherries, raspberries, or blueberries), (2) unmanaged, wild fruit, and (3) for a standardized sample, we used banana traps. The sampling of the different fruit types served to capture the range of hosts available for *D. suzukii* and to account for the possible effects pesticide use and residues in the managed fruit samples may have on the fitness of flies. For the managed fruit collection, 0.5 kg of conventionally grown (i.e., pesticide‐sprayed) fruit was picked from cultivated plants and fallen fruit from the ground. For the unmanaged fruit samples, 0.5 kg of fruit was collected from brambles and other wild soft fruit‐bearing plants (i.e., blackberries or pokeweed) that were located along field edges. Each fruit sample was collected by hand and from a minimum of three different plants. For the banana traps, one halved banana was placed in a 454 mL lidded red solo cup with several holes drilled into the bottom and lid to allow flies to enter and rainwater to leave. Wires were threaded through holes at the top of the cup and used to hang the traps from trees located along the edge of orchards. At each site, three banana traps were placed and left for 48 h to be infested by fruit flies. An eighth field site located in Corvallis, Oregon, was sampled similarly in August and September 2022, and the collected fruit was mailed to MSU to rear out fruit flies and for subsequent resistance assays.

**TABLE 1:** Study locations, site characteristics, and sampling dates. Site ID refers to locations as shown in Figure 1. C = Central, E = East, EC = East Central, N = North, SW = Southwest, W = West, WC = West Central.

Site ID	Location	Coordinates		Crop type	Management	Sampling date	
		Latitude	Longitude			Aug	Sep
1OR	Corvallis, OR	44.6102	−123.2252	Mixed Fruits and Veg.	Organic	8/16/2022	9/14/2022
2N	Bingham, MI	44.8832	−85.6751	Mixed Fruits	Conventional	8/11/2022	9/19/2022
3W	Fennville, MI	42.5946	−86.1555	Mixed Fruits	Conventional	8/8/2022	9/14/2022
4SW	Niles, MI	41.7881	−86.3046	Mixed Fruits	Conventional	8/8/2022	9/14/2022
5WC	Clarksville, MI	42.8735	−85.2587	Mixed Fruits and Veg.	Conventional	8/8/2022	9/24/2022
6C	East Lansing, MI	42.6749	−84.4898	Mixed Fruits and Veg.	Organic	8/12/2022	9/19/2022
7EC	Flint, MI	43.0240	−83.6753	Mixed Fruits	Conventional	8/9/2022	9/16/2022
8E	Washington, MI	42.7727	−83.0185	Mixed Fruits	Conventional	8/9/2022	9/16/2022

### 2.3 Resistance Assays

Fruit from each site and sample type was placed in separate plastic containers (25 × 19 × 25 cm, PrepNaturals) in an incubator (25°C ± 2°C, 70% RH, 16L:8D) for 10 days. Emerging adult *D. suzukii* were identified based on morphology, separated from other fruit fly species, and transferred to drosophila vials (2.5 × 9.5 cm) (Genesee Scientific, San Diego, CA, USA) containing 6 mL standard DSSC cornmeal diet. Adult flies were allowed to mate and oviposit in the artificial diet for 48 h. Three days after the adult flies were removed, 50 second instar larvae were extracted from the artificial diet from each sample. The 50 larvae were divided into five replicates of 10 larvae each for each location and sample type combination.

The larvae for each replicate were placed atop a halved blueberry to prevent dehydration inside a 60 × 15 mm plastic Petri dish (Falcon, Corning, NY, USA). Each Petri dish then received one female *G. brasiliensis* wasp that had been isolated with a single male in a drosophila vial for 24 h to mate. The female wasp was left in the Petri dish for 4 h to parasitize *D. suzukii* larvae. Because of the limited availability of *G. brasiliensis* females, we had to reuse the same individuals for multiple rounds of parasitism. Individual females were first used to parasitize *D. suzukii* larvae from each of the eight field sites of the five replicates of the banana traps (*n* = 40 females). The same females were then moved onto the *D. suzukii* samples collected from the managed fruit to parasitize for 4 h and after that onto the samples from unmanaged fruit for 4 h.

The *D. suzukii* larvae were left in the halved blueberries for 72 h to allow the effects of parasitism and encapsulation to become visible. The larvae were then removed and examined under a microscope (Nikon SMZ1000, 10–80× magnification) for evidence of parasitism (the presence of one parasitoid egg per fly larva) and encapsulation of the parasitoid egg that is indicated by the darkening of the egg as it is encased in melanized host tissue and results in the death of the parasitoid. Each parasitized host had at most one egg. This process allowed us to see the results of our resistance assays more quickly than if we had waited to examine *D. suzukii* adults after they emerged. For most larvae, these signs were visible under a microscope with sufficient light, but if no evidence of parasitism or encapsulation was immediately clear, larvae were dissected with ophthalmological spring scissors (Fine Science Tools). In each replicate, the number of larvae that were parasitized and the number of encapsulated parasitoid eggs were recorded.

### 2.4 Statistical Analyses

All statistical analyses were performed using R software 4.2.3 (R Core Team 2013). Generalized linear mixed effects models (GLMER) from the lme4 package were constructed to determine the impact of fixed effects on our dependent variables, while comparison between effects was performed using the *emmeans* package (Bates et al. 2015; Lenth et al. 2021). All post hoc pairwise comparisons were performed using the *emmeans* package with Tukey adjustment (Lenth et al. 2021). The dependent variables (parasitism rate and encapsulation rate) were treated as binomial outcomes since the proportional values were bounded between 0 and 1. Parasitism was calculated for each female by dividing the number of parasitized hosts that included both encapsulated and nonencapsulated eggs (*n*_p_) by the number of hosts offered (*n*_0_ = 10) to create a parasitism rate (PR): PR = *n*_p_/*n*_0_. Encapsulation rate (ER) was calculated by dividing the number of encapsulated eggs in parasitized hosts (*n*_e_) by the number of parasitized hosts (*n*_p_): ER = *n*_e_/*n*_p_.

Parasitism rate and encapsulation rate of *D. suzukii* larvae collected at different locations and at different times were compared using GLMER. In these models, location (the eight field sites), month (August or September), and the interaction between the location and month were included as fixed effects. Because the same females were reused for parasitism sequentially on the three different fruit types (banana trap first, managed fruit second, and unmanaged fruit third) from each site in both months sampled, fruit type nested within the month was included as the random effect.

## 3 Results

### 3.1 Parasitism Rate

**FIGURE 2:**
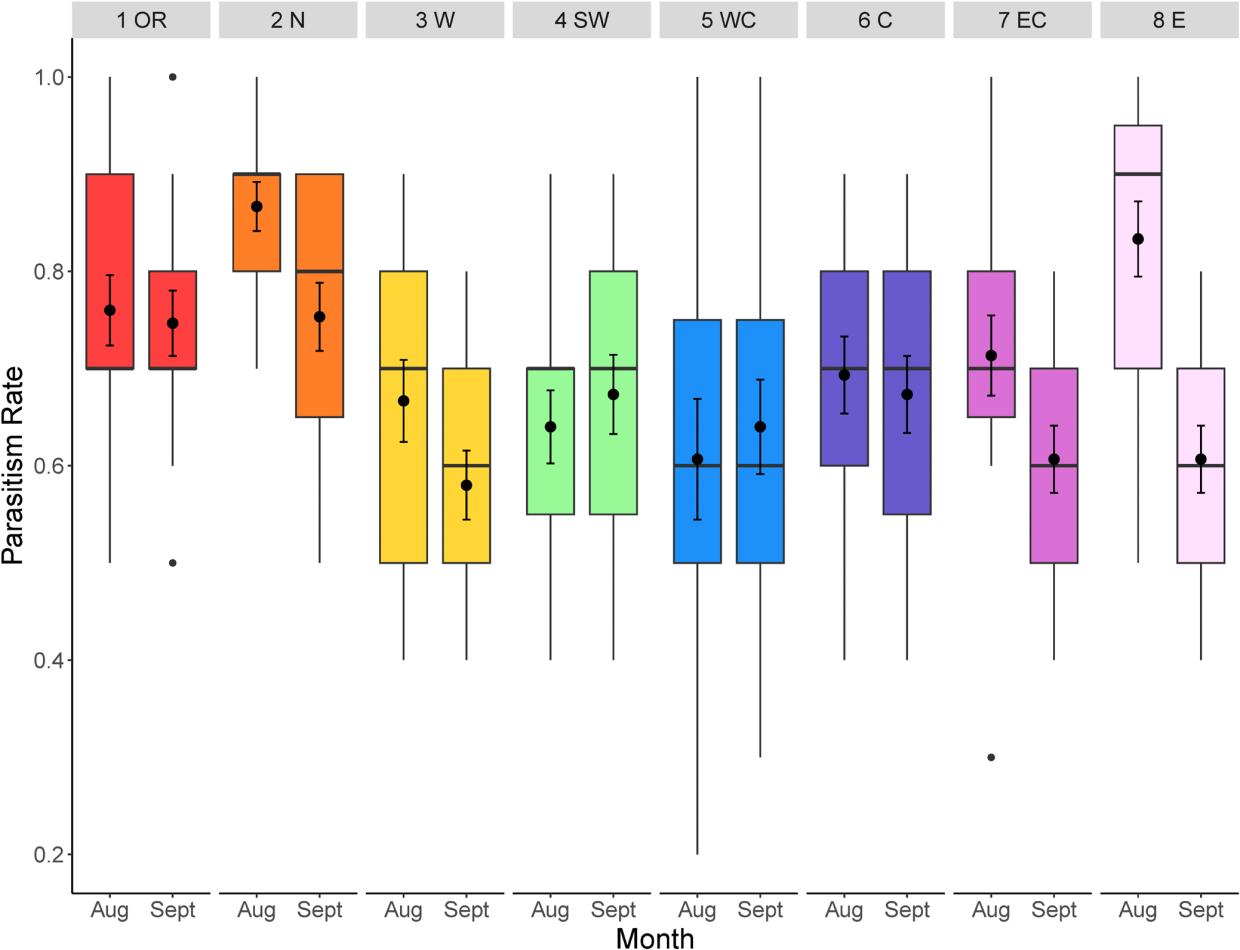
Parasitism rates of *Ganaspis brasiliensis* by eight populations of *D. suzukii* (1 population in Oregon—1OR and seven populations in Michigan—2N—8E) sampled in August and September. The numbers on top indicate the sampling locations as shown in Figure 1. Dots indicate outlier observations; the horizontal line indicates the median, with the box representing the interquartile range, and vertical lines are 1.5 times the interquartile range. Means and standard errors are shown within each box plot.

Parasitism rate of *D. suzukii* by *G. brasiliensis* differed significantly among samples from different locations (*F*_7,230_ = 22.73, *p* < 0.001) and between months (*F*_1,229_ = 10.69, *p* < 0.001). The northernmost site in Michigan (2N in Figure 1) had the highest parasitism rate (mean parasitism ± SE; 82% ± 2.49%) that was significantly higher than the westernmost (3 W) (64.6% ± 3.51%), southwestern (4SW) (68.5% ± 3.31%), and east central (7EC) (68.1% ± 3.36%) locations (all pairwise comparisons: *p* < 0.05) (Figure 2). Overall, parasitism rates of *D. suzukii* tended to be higher for collections made in August (76.1% ± 1.48%) than those made in September (68.5% ± 1.67%) (pairwise comparison: *p* = 0.0007). These results were mostly consistent within locations (month × location interaction: *F*_7,222_ = 11.01, *p* = 0.138).

### 3.2 Encapsulation Rate

**FIGURE 3:**
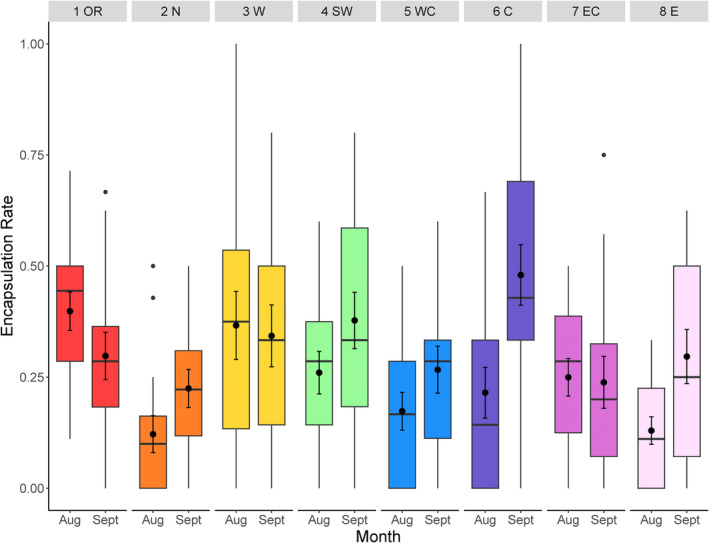
Encapsulation rates of *G. brasiliensis* by eight populations of *D. suzukii* (1 population in Oregon—1OR and seven populations in Michigan—2N—8E) sampled in August and September. The numbers on top indicate the sampling locations as shown in Figure 1. Dots indicate outlier observations; the horizontal line indicates the median, with the box representing the interquartile range, and vertical lines are 1.5 times the interquartile range. Means and standard errors are shown within each box plot.

Encapsulation rates (resistance) differed among locations where *D. suzukii* was collected from (*F*_7,230_ = 30.88, *p* < 0.0001) and between months (*F*_1,229_ = 9.67, *p* = 0.0018). Encapsulation rates ranged from 11.5% ± 2.8% in August at the northern site (2N) to 47.5% ± 4.97% in September at the central Michigan site (6C) (Figure 3). Overall, the northernmost site that had the highest parasitism rate showed the lowest mean encapsulation rate across months (16.1 ± 2.41%). Encapsulation rates of the northernmost site were significantly lower than that of the Oregon (33.4% ± 3.16%), the westernmost (3W) (31.1% ± 3.39%), the southwest (4SW) (30.2 ± 3.29), and the central Michigan sites (6C) (32.4% ± 3.45%) (all pairwise comparisons: *p* < 0.05) (Figure 3). The month of sampling affected the resistance levels, with *D. suzukii* collected in September (29.9% ± 1.66%) demonstrating higher encapsulation rates than the August samples (22.3% ± 1.46%) (pairwise comparison: *p* = 0.0006). However, the higher encapsulation rates later in the season were not consistent across sites (location × month interaction: *F*_7,222_ = 25.298, *p* = 0.0007). Of the eight sites, only the central Michigan (6C) location showed a significantly higher resistance in the September samples compared to the August samples (pairwise comparison: *p* < 0.0001) (Figure 3) where the mean encapsulation rates were 20.2% ± 3.94% in August and 47.5% ± 4.97% in September.

## 4 Discussion

We found geographic and temporal differences in the levels of resistance of invasive *D. suzukii* populations in North America against the specialist larval parasitoid *G. brasiliensis* that is being released as a biological control agent across North America and Europe. Encapsulation rates reached up to 48% locally and tended to increase as the season progressed. These results can have implications for the incipient classical biological control program that uses mass releases of *G. brasiliensis* and may cause spatial and temporal variations in control success.

Encapsulation rates of *G. brasiliensis* eggs by *D. suzukii* ranged between 12% and 39% in August and 22%–48% in September regionally. A direct comparison of these results with other studies is difficult because of the different methods used to assess encapsulation. We evaluated parasitized larvae to assess the parasitism and encapsulation rates (Kacsoh and Schlenke 2012), while the two other studies that assessed the outcome of parasitism by *G. brasiliensis* examined the presence of black capsules in adult flies (Daane et al. 2021; Girod et al. 2018). The study using *D. suzukii* populations from California and *G. brasiliensis* from South Korea found around 5% encapsulation rates (Daane et al. 2021). The other study that used the same G1 strain of *G. brasiliensis* from Japan as used here and *D. suzukii* from mixed populations in Switzerland found 15.7% of encapsulation rates in blueberries (Girod et al. 2018). Even though our study does not account for mortality that could occur during development, the measured encapsulation rates are similar for Swiss *D. suzukii* (Girod et al. 2018) and the northernmost site in Michigan where the mean encapsulation over 2 months was 16.1%. However, in the other Michigan locations that are warmer and in Oregon, encapsulation rates were generally higher (21%–32%). These levels of resistance against *G. brasiliensis* are comparable to another coevolved larval parasitoid, *Asobara japonica* (Belokobylskij) (Hymenoptera: Braconidae), that has a much broader host range than the G1 strain of *G. brasiliensis* and had 6%–26% encapsulation rates by multiple strains of *D. suzukii* (Poyet et al. 2013). In contrast, the European parasitoid *L. heterotoma* (Thomson) that did not have a coevolutionary history with *D. suzukii* was encapsulated at rates of 59%–87% (Poyet et al. 2013). The strength of resistance correlated with hemocyte levels in the flies that were twice as high in three invasive *D. suzukii* populations from France as in two native populations from Japan (Poyet et al. 2013). This indicates that the introduced populations of *D. suzukii* have maintained relatively high levels of defenses against parasitism in France (Poyet et al. 2013), and at least some of the sampled populations in Michigan and the one from Oregon also show encapsulation rates of over 30%. There is evidence from other drosophilids that the maintenance of high hemocyte load can only be costly in competitive environments. For example, when food is plentiful, there was no cost to resistance in three *Drosophila* species (McGonigle et al. 2017). It is possible that *D. suzukii* can maintain high levels of defensive compounds without trade‐offs because it currently experiences relatively low competition and high resource availability due to its ability to attack still ripening fruit with its serrated ovipositor (Poyet et al. 2013). In contrast, native drosophilids in Europe and North America are restricted to attacking overripe and fallen fruit and thus occupy different niches than *D. suzukii* (Asplen et al. 2015; Lee et al. 2019).

We found great variation in resistance regionally and temporally in *D. suzukii* with lows of 11% encapsulation in northern Michigan and highs of 39% in Oregon in August and 48% in central Michigan in September. Similar variation in parasitoid resistance has been seen in other *Drosophila* species which have demonstrated population‐level differences in encapsulation rates based on geographic location (Kraaijeveld and Van Alphen 1995; Kraaijeveld and Godfray 1999; Dubuffet et al. 2007). For example, *D. melanogaster* resistance to one of its native generalist larval parasitoids, *A. tabida*, is the highest in central Europe (40%–60% encapsulation rates) and lower in northern and southern Europe (Kraaijeveld and Godfray 1999). Seven geographically distinct populations of *D. yakuba*, a fly species native to Africa, also show varying encapsulation rates of its native larval parasitoid *L. boulardi* that range between 6% and nearly 98% regionally (Dubuffet et al. 2007). In native host–parasitoid communities where multiple parasitoid and host species interact, the regional difference in community structure can be the most important driver of geographic differences in host defenses (Kraaijeveld and Godfray 1999). Introduced species can also experience regional differences in host–parasitoid community structure, and thus variable selection pressures to maintain defenses, because of variation in attack rates by native parasitoids in the introduced range and by unintentionally introduced coevolved parasitoids from the invasive species' native range. Recent and widespread detections of the coevolved larval parasitoid *L. japonica* both in Oregon and Michigan and eight other states indicate that these parasitoids have probably been around for years but went undetected (Gariepy et al. 2024). This means that while initially mostly native generalist pupal parasitoids attacked invasive *D. suzukii* populations (Lee et al. 2019), selection pressures for increased resistance could have mounted regionally as *L. japonica* spread across the landscape. The presence of *L. japonica* may have partly accounted for some of the higher encapsulation rates we found. For example, in western Michigan where *L. japonica* was monitored and detected (Gariepy et al. 2024), encapsulation rates were 31% (3W) and 35% (4SW) in September. However, the distribution of *L. japonica* is unknown in other parts of Michigan, limiting the inferences we can make. Thus, it appears that *D. suzukii* initially experienced some form of enemy release upon introduction to North America but over time coevolved natural enemies have spread across the landscape with likely patchy distribution and variable population densities that could have mediated geographic variation in the resistance of *D. suzukii* populations.

In addition to variations in the presence and density of native and introduced parasitoids, abiotic factors could also influence the resistance levels of *D. suzukii* populations. Our results suggest that temperature might be one of the factors affecting resistance since the northernmost site in Michigan had the lowest encapsulation rate. This site is the coldest as it is located over 300 km to the north of other sites in Michigan, and lower temperatures have been associated with lower encapsulation ability in insect host–parasitoid interactions (Blumberg 1991; Fellowes, Kraaijeveld, and Godfray 1999). There were also temporal differences in the resistance of *D. suzukii* populations that show the opposite pattern, that is, somewhat increasing encapsulation rates later in the season. However, this difference was only significant between August and September for the central Michigan site (6C in Figure 1), and *D. suzukii* collected in Oregon showed the reverse pattern of lower encapsulation in September than in August. Thus, it is likely that temperature effects on host resistance are not linear and are mediated by complex interactions between the host, their parasitoids, and the environment (Thomas and Blanford 2003).

In addition to the community structure and abiotic conditions, the genetic structure of *D. suzukii* populations may also influence resistance; however, there is currently no evidence for this to be the primary factor mediating differences in encapsulation rates. Multiple studies found large‐scale genetic differentiation between eastern and western North American populations of *D. suzukii* but not within local populations within regions (Adrion et al. 2014; Fraimout et al. 2017; Lewald et al. 2021; Nelson et al. 2023).

Parasitism rates of *D. suzukii* populations also differed regionally and over time; however, parasitism tended to show opposite patterns than encapsulation. For example, the northern site that demonstrated the lowest encapsulation rates showed the highest rates of parasitism. Similarly, the easternmost site that had low encapsulation rates had relatively high parasitism rates. While the encapsulation rate increased from August to September, parasitism rate of the same populations decreased between those months. These patterns suggest that *G. brasiliensis* may be able to assess the resistance ability of larvae and lays fewer eggs in better defended individuals. Such correlations have been shown with the parasitoid *A. tabida* that tended to reject *D. melanogaster* larvae that had high resistance to parasitism (Kraaijeveld, Nowee, and Najem 1995). Similarly, in 16 different Lepidopteran species, caterpillars with the highest levels of resistance had the lowest levels of parasitism (Smilanich, Dyer, and Gentry 2009).

The above results indicate that there can be differences in the regional outcome of host–parasitoid interactions that may influence the efficacy of *G. brasiliensis* as a biological control agent of *D. suzukii*. At locations where *D. suzukii* have relatively low resistance, the impact of biocontrol may be larger initially, while increased or geographically variable levels of resistance could render biocontrol less successful or more variable regionally. Our results could help to assess the mechanisms that may underlie biocontrol success or failure, and different management approaches may be recommended based on spatial differences in the resistance of *D. suzukii* populations. For example, higher parasitoid releases may be necessary at locations that show relatively high levels of *D. suzukii* resistance to increase parasitoid pressure and to ensure that *G. brasiliensis* populations can maintain high densities despite their lower success of development. The higher population sizes and densities could help *G. brasiliensis* to maintain genetic diversity and adaptive potential and to evolve higher virulence over time. An alternative approach may be to release *D. suzukii* flies with low levels of resistance at sites that showed high encapsulation rates to reduce the overall resistance by mixing populations. In any case, the baseline data collected here will be valuable to further explore the eco‐evolutionary dynamics of host–parasitoid interactions following the release of *G. brasiliensis* across Michigan.

## Conflicts of Interest

The authors declare no conflicts of interest.

## Data Availability

Data for this study are available at https://doi.org/10.5061/dryad.4xgxd25jg
